# Adult-Onset Neuronal Ceroid Lipofuscinosis: *CLN5* Variant Presenting as Focal Dystonia

**DOI:** 10.5334/tohm.941

**Published:** 2024-11-04

**Authors:** Karri Madhavi, Rukmini Mridula Kandadai, Sruthi Kola, Rupam Borgohain, Rajesh Alugolu, Vvsrk Prasad, Bevinahalli N. Nandeesh, Periyasamy Govindaraj

**Affiliations:** 1 Department of Parkinson’s and Movement Disorders Research Centre (PDMDRC), Citi Neuro Centre, Banjara Hills, Hyderabad, Telangana, India; 2 Department of Neuropathology, National Institute of Mental Health and Neurosciences (NIMHANS), Bengaluru-29, Karnataka, India

**Keywords:** Dystonia, CLN5 variant, Adult-onset Neuronal ceroid lipofuscinosis

## Abstract

**Background:**

Neuronal ceroid lipofuscinosis (NCL) is a rare hereditary lysosomal storage disorder causing neuronal loss and progressive neurodegeneration. *CLN* variants cause varied phenotypic presentations.

**Case report:**

A 49-year-old male presented with late adult-onset progressive focal right lower limb dystonia. Imaging showed cerebellar atrophy, and genetic testing was positive for the *CLN5* variant (c.826T > C; p.Phe276 Leu) with uncertain significance. Skin biopsy suggested NCL, which made us consider the variant pathogenic, leading to novel phenotypic presentation.

**Conclusion:**

Isolated focal dystonia has not been reported as an initial presentation in ANCL. Early genetic testing and periodic clinical assessments are advisable for better management and prognostication.

## Introduction

Neuronal ceroid lipofuscinosis (NCL) is a rare progressive neurodegenerative disorder affecting children and adults. It is a lysosomal disorder whose inheritance is mainly autosomal recessive but can also be autosomal dominant. It is characterised by intraneuronal accumulation of auto-fluorescent ceroid pigment, causing neuronal loss and progressive neurodegeneration. The disorder has a broad phenotypic spectrum that includes visual disturbances, seizures, movement disorders, cognitive impairment, and behavioural abnormalities. Adult-onset NCL (ANCL) has two phenotypic forms: Kufs type A and Kufs type B [1].Movement disorders reported include myoclonus, ataxia, tremors, and rarely parkinsonism. The genes reported in ANCL are *CLN6, DNAJC5, CTSF, CLN1, CLN5, ATP13A2*, and *GRN* [2]. We present a case of focal dystonia secondary to the *CLN5* variant, where the work in genetic testing in diagnosing and managing such rare neurodegenerative conditions is crucial and highly valued.

## Case description

**Figure 1:**
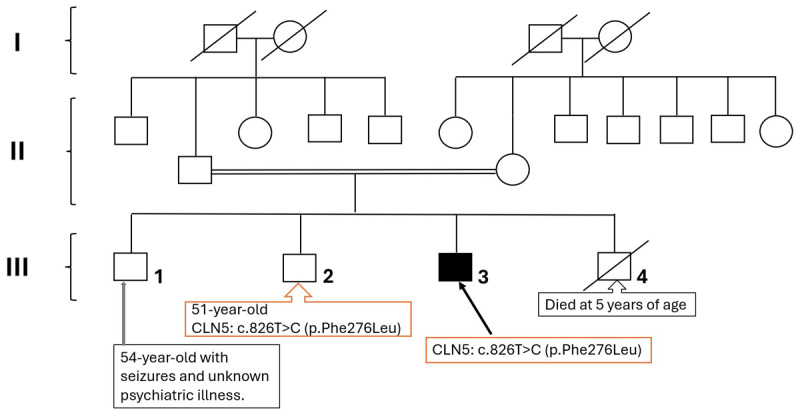
Pedigree chart of the patient.

A 49-year-old male presented with pain in his right foot followed by posturing in the great toe and foot, which started three years back. It gradually progressed, involving the entire right lower limb for the past three months. There was no history of seizures, vision impairment, memory and behavioural disturbances. He is the third child of four (Figure 1, III-3), born to fourth-degree consanguineous parents (first cousins), with normal birth and developmental milestones and no significant perinatal events. There was a history of poliomyelitis at five years of age in III-1 with history of seizures and an undefined unknown psychiatric illness (evaluated elsewhere with details unknown). The patient’s younger brother, III-4, died at the age of 5 years from an unknown systemic illness (Figure 1).

**Figure 2:**
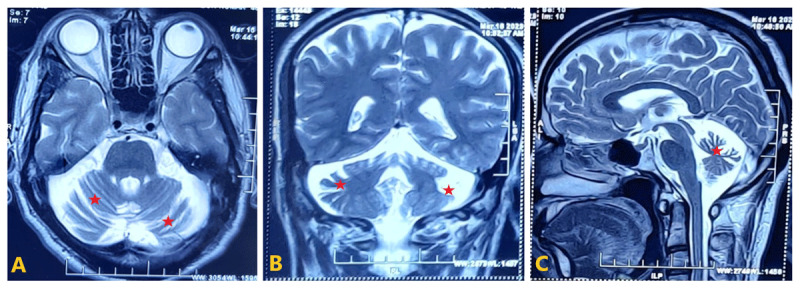
Magnetic resonance imaging (MRI) images of the brain – T2 weighted images (1B- Axial, 1C – Coronal, 1D – Sagittal): showing cerebellar atrophy [red star] with normal cerebral parenchyma, basal ganglia and brain stem with no evidence of other structural lesions.

Clinically, ocular examination showed normal eye movements with spontaneous horizontal nystagmus at the central position, further confirmed by video nystagmography (Video). His visual acuity was normal. The tone and power were normal. Deep tendon reflexes were normal. Extrapyramidal examination showed dystonic posturing of the right leg partially reduced with wearing footwear (sensory trick). The speech was normal. The finger-nose and the knee-heel test were impaired. Gait showed dystonic posturing of the right leg. Dystonic posturing was in the form of flexion at the hip and knee joint with dorsiflexion of the ankle joint associated with external rotation of the leg. There was no spread of dystonia to other limbs or body parts while walking or at rest. Dystonia resolved entirely while walking backwards (Video). Dystonia was also resolved entirely at rest when lying in the supine position, whereas, in a sitting posture, there was dorsiflexion of the ankle and toes was noticed, which was reduced with wearing footwear – suggesting the presence of exteroceptive sensory trick (geste antagoniste). Tandem gait was impaired, and Romberg’s sign was negative. Montreal cognitive assessment (MoCA) score was 30/30. Blood parameters were tested to identify treatable and reversible causes of adult-onset lower limb focal dystonia. They included renal and liver metabolic parameters, serum electrolytes with magnesium levels, serum parathormone levels, copper and ceruloplasmin levels, and autoimmune and paraneoplastic panels, which were unremarkable. Brain magnetic resonance imaging (MRI) showed cerebellar atrophy (Figure 2A, B, C) with normal cerebral parenchyma without thinning of white matter and with no evidence of structural lesions. No evidence of blooming was noted in susceptibility-weighted imaging. There were no T1 hyperintensities or T2 hypo-intensities in the basal ganglia or brainstem. Whole exome sequencing showed a homozygous missense variant in exon 4 of *CLN5* (NM_006493.4): c.826T > C (p.Phe276 Leu) of uncertain significance. His brother III-2, who is 52 years old, on genetic testing, was found to harbour the heterozygous variant of uncertain significance of *CLN5* (NM_006493.4): c.826T > C (p.Phe276 Leu). He had no history of defined similar or other neurological illnesses. Whereas III-1, who had other neurological illnesses as described before, was advised to undergo genetic testing for the same variant but was unwilling to get the test done due to personal reasons.

**Video:** A video demonstrating the patient with **A**. Video nystagmography showing spontaneous horizontal nystagmus at central gaze. **B**. Gait – Right lower limb dystonic posturing on walking (hip flexion, knee flexion, and ankle dorsiflexion with external leg rotation). After the first dose of botulinum toxin, there was mild improvement in the dystonic posture of the leg. On backward walking, there is no dystonia in the right lower limb. **C**. Demonstration of cerebellar signs – finger-nose and knee-heel tests were abnormal with a positive rebound phenomenon.

**Figure 3:**
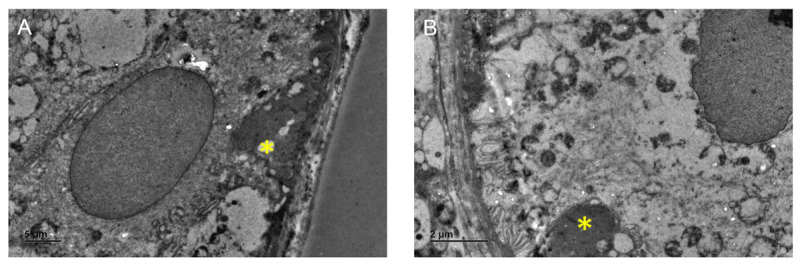
The electromyography of the skin showing (A & B) epithelial cells of the eccrine glands with basal granular osmiophilic deposits (GROD) [yellow asterisk]* (Magnification: A-X1500 & B – X2000).

Skin biopsy on light microscopy showed no distinctly seen inclusions. Hence, the sample was processed for electron microscopy (2.5% glutaraldehyde was used as a fixative), and toluidine blue-stained semithin sections showed a portion of dermal collagen with eccrine glands. Ultrathin sections of electron microscopy showed eccrine glands with scattered granular osmiophilic inclusions with vacuolar changes in a few mitochondria, with the rest of the organelles, including mitochondria, being intact, suggestive of NCL (Figure 3).

He was initially started on 300 mg of levodopa and 75 mg of carbidopa per day in three divided doses. He initially found that it had a transient response in the form of mild improvement in dystonic posturing, where he described being able to walk for longer distances with less posturing, though not resolved entirely. Later, it was stopped after a month as he had no further sustained beneficial response with levodopa. He received two doses of botulinum toxin type A for his right leg dystonia. For the first dose, he received 80 units (60 units for hamstrings and 20 units for tibialis anterior). After four months, he received the second dose of 120 units (60 units for hamstrings, 20 units each for peroneus tertius, pectineus and tibialis anterior muscles). He showed a mild response to botulinum toxin while being able to walk comparatively longer distances than the distance before receiving the injections (Video). Currently, he is on baclofen, tetrabenazine, clonazepam and trihexyphenidyl medications, with two doses of botulinum toxin doses taken.

## Discussion

Lysosomal storage disorders (LSDs) are caused by single-gene defects encoding for lysosomal proteins, and clinical phenotypes depend upon substrate accumulation and organ involvement. The classification based on the substrate is a mere convenience as there may be an overlap of the enzyme substrates [3,4]. The current understanding and advances have shown its association with neurodegenerative conditions like Parkinson’s disease [5]. The autophagy-lysosomal pathway is involved in both conditions and contributes to neurodegeneration [6]. With this background, diagnosing atypical phenotypic presentations may be difficult. On the other hand, more than fifty variants have been identified implicating LSDs. Still, some cases were diagnosed based on apparent clinical criteria where variant testing was either not implicated in the disease or may remain elusive [5].

Within the LSDs, NCLs are a group of disorders comprising at least 13 distinctive types based on the gene variant and age of manifestation, indicated as CLN1 through CLN14 (with CLN9 type caused by *CLN5* variants) [7]. About 5% of *CLN* mutations are associated with ANCL (ageing between 17 to 49 years), and often presented phenotypically like classical childhood forms. However, atypical presentations have been reported [8]. NCL genes are allelic with variants contributing to neurodegenerative disorders like Alzheimer’s disease, Parkinson’s disease and frontotemporal dementia [9]. Therefore, diagnosis of ANCL is often challenging as it mimics other neurodegenerative conditions, and extra-neural pathological diagnostic yield is often limited and misinterpreted [10].

*CLN5* mutation causes NCL in the late infantile stage and rarely in adulthood. Ataxia, myoclonus and progressive cognitive impairment are the most common presentations noted in the *CLN5* variant or any other ANCL [8]. Only one case report has been reported of *CLN5* variant presenting with atypical parkinsonism with dystonia [11]. We identified a homozygous missense variant, c.826T > C p.Phe276 Leu at exon 4 in our patient, which differs from the *CLN5* variants reported in ANCL. (Table 1). This variant’s gnomAD frequency was absent, with a combined annotation-dependent depletion (CADD) score of 28.6 and a rare exome variant ensemble learner (REVEL) score of 0.052. The CADD score being more than 20 indicates that the variant might alter the protein’s composition and physicochemical properties. Multiple lines of computational evidence (Polyphen – probably damaging, SIFT (sorting intolerant from tolerant) – damaging and MutationTaster – disease-causing) predicted this variant’s damaging effect on the protein structure and function. This variant has not been reported so far, and with the additional skin biopsy findings in our case, we concluded that this variant might have caused the disease with a different phenotypic presentation. To our knowledge, this is the first case report of ANCL presenting as isolated focal dystonia as an initial presentation that later developed subtle ataxia. This presentation might be attributed to the novel genetic variant in *CLN5*, as reported in this case. ANCL is considered one of the differentials in adult-onset lower limb dystonia associated with other neurological signs. Here, we suggest that isolated progressive focal dystonia can be a phenotypic presentation in ANCL, where early genetic testing and periodic clinical assessments are recommended to understand the evolution of the phenotypic presentation, which might fulfil the diagnostic criteria.

**Table 1:** Reports of *CLN5* mutations in adult-onset neuronal ceroid lipofuscinosis (ANCL).


PAPER	AGE OF ONSET (YEARS)/GENDER	PHENOTYPE	VARIANT

Current study	46/Male	Focal dystonia	Homozygous variant of exon 4 of *CLN5* (c.826T > C, p.Phe276 Leu)

Sleat DE et al [12].	20/Female	Unknown (postmortem study)	Two missense variants of *CLN5* (c.377G3 A, p.Cys126Tyr) (c.1121 A3G, p.Tyr374Cys)

Xin W et al [13].	17 (2 patients)	Cognitive regression and visual loss, seizure and motor difficulty	Compound heterozygous variant of exon 2 of *CLN5 (*c.377GA, p.Cys126Tyr) and exon 4 of *CLN5* (c.1121 AG, p.Tyr374Cys)

		Motor difficulty, seizure, visual loss and cognitive regression	Exon 4 of *CLN5* (c.1121 AG, p.Tyr374Cys)

Lange LM et al [11].	27/Female	Early-onset parkinsonism with atypical signs like dementia, dystonia, ataxia, and visual impairment	Two heterozygous variants of *CLN5* (c.486 + 4dupA and c.575 A > G, p.Asn192Ser).

Mancini, C. et al [8].	50/Female	Gait instability, dysarthria, and a mild cognitive deficit	Homozygous variant in exon 4 of *CLN5* (c.935G > A; p.-Ser312 Asn)

	56/Male	Gait problems and dysarthria	

